# Effects of Different Rates of Poultry Manure and Split Applications of Urea Fertilizer on Soil Chemical Properties, Growth, and Yield of Maize

**DOI:** 10.1155/2020/4610515

**Published:** 2020-08-04

**Authors:** Aruna Olasekan Adekiya, Oluwaseyi Iyabo Ogunboye, Babatunde Sunday Ewulo, Adeniyi Olayanju

**Affiliations:** ^1^College of Agricultural Sciences, Landmark University, P. M. B. 1001, Omu-Aran, Kwara State, Nigeria; ^2^Department of Crop, Soil and Pest Management, Federal University of Technology, Akure, Ondo State, Nigeria; ^3^Department of Agricultural and Bio-System Engineering, College of Engineering, Landmark University, P. M. B. 1001, Omu-Aran, Kwara State, Nigeria

## Abstract

During integrated nutrient management involving poultry manure (PM) and urea fertilizer (UF) for maize (Zea mays L.), it is necessary to investigate the best time to apply UF that will optimize soil chemical properties, growth, and yield of maize. Hence, studies were carried out to investigate the effect of different rates of PM and single and split applications (SA) of UF on soil chemical properties, growth, and yield of maize. The treatment involved three levels of PM (0.0, 4.0, and 8.0 t*·*ha^−1^) and four sets of periods of UF: (i) 0 kg N ha^−1^ (control), (ii) 120 kg N ha^−1^ applied at planting (AP), (iii) two SA of 120 kg N ha^−1^ (90 kg N ha^−1^ applied AP + 30 kg N ha^−1^ at thirty days after planting (DAP)), and (iv) three SA of 120 kg N ha^−1^ (60 kg N ha^−1^ applied AP + 30 kg N ha^−1^ thirty DAP + 30 kg N ha^−1^ at tasseling). The 12 treatments were arranged in a randomized complete block design and replicated three times. PM and UF alone and integrating UF with PM improved soil chemical properties, growth, and yield of maize compared with the control. SA of UF three times (60 + 30 + 30) had the most improved soil chemical properties, growth, and yield of maize. Results also showed that maize yielded higher under UF integrated with PM compared with their sole forms. Application of 60 + 30+30 with 8 *t* ha^−1^PM (60 + 30+30 + 8 *t* ha^−1^PM) or with 4 *t* ha^−1^PM treatments showed the highest growth and yield of maize, but due to the bulkiness, handling challenges, and cost of PM, 60 + 30+30 + 4 *t* ha^−1^PM is recommended. Therefore, for better growth and yield of maize, after the initial application of PM, UF application should be split-applied in accordance with plant growth and the pattern of uptake to avoid losses by leaching and therefore ensured that N level in the soil is high at the critical stage of N demand.

## 1. Introduction

Maize (*Zea mays* L.) is an important cereal crop worldwide especially in developing countries where it is eaten as a staple food. In Nigeria, every part of the plant is important. The grains can be consumed, roasted, baked, fried, ponded, and porridges. It is also an important source of industrial products like corn sugar, porridges, beverages, bread, and snacks [1]. Maize is a major component of livestock feed and it is palatable to poultry, cattle, and pig [2]. The stalk, leaves, and immature ears are cherished by livestock.

Due to its importance in Nigeria, the area under maize production has increased over the years from about 438,000 ha in 1981 to over 3.3 million ha in 2009 [3]. However, despite the increase in area under maize production in Nigeria, the amount produced cannot meet the demand for it. This was related to low yield, as the yield of maize in Nigeria is generally low, being about 1.4 *t* ha-1 compared with the world average of 5.5 *t* ha^−1^ and USA of 9.5 *t* ha^−1^ [4]. One of the major problems of the low harvest of maize in Nigeria is that of inherently poor soil. Poor soil fertility is the major cause behind low crop yield including maize. One of the ways of increasing the yield per unit area of soil is by the addition of external input including organic and chemical fertilizers.

Maize is a nutrient demanding crop and therefore adequate and balanced nutrient supply is important in its growth and production. The use of chemical fertilizer has been reported [5] to increase crop yields, but in Nigeria, its use is limited by high cost, scarcity during the time of its need (planting season), soil acidity, and nutrient imbalance. Because of these, the use of organic manure like poultry manure (PM) was found useful in increasing crop production. PM is cheap, readily available at all times, environmentally friendly, and also has a residual effect and ability to improve soil structure compared with chemical fertilizers. It contains N, P, and K and other essential elements [6]. PM application increases soil N by more than 53%, while exchangeable cations are also increased significantly upon application [7]. The rate of PM applied may also influence the amount of nutrient released (soil chemical properties) growth and yield of maize. However, PM is limited by the large quantities required for large scale maize production [8]. Therefore, to avert this problem, there is strong advocacy for integrating organic and inorganic fertilizers [9, 10]. For maize that requires large amounts of N for its growth and yield, the use of PM with urea fertilizer can be very beneficial. A positive interaction has been reported to occur between organic manure and urea as N source [11]. Mani et al. [12] reported that using indigenous available organic nutrient source can enhance the efficiency and reduce the quantity of chemical fertilizer required. Apart from enhancing nutrient use efficiency, integrated nutrient use also maintains soil health, enhances yield, and reduces cost of production [12].

For integrated nutrient management in maize cultivation, PM is usually applied to the prepared soil two weeks before planting maize [9] to allow the mineralization of the PM. Urea may be applied to maize farms in different growing stages. Delaying or early application of urea to maize plants may have an implication on soil chemical properties, growth, and yield of the crop. Many researchers have suggested that N should be applied at the time it is needed by the crop [13, 14]. However, it is necessary to find the best time for urea application in maize to increase nitrogen use efficiency [13]. During integrated nutrient management involving PM and urea fertilizer for maize, it is, therefore, necessary to investigate the best time during the growth of the crop to apply urea fertilizer that will optimize soil chemical properties, growth, and yield of maize. Therefore, the objectives of this study were to investigate the effect of different rates of PM and single and split applications of urea fertilizer on soil chemical properties, growth, and yield of maize.

## 2. Materials and Methods

### 2.1. Experimental Site

The experiments were carried out in July 2014 at two locations (Site I and Site II) in Osogbo Agricultural-Farm Settlement, Osogbo, Osun State, Southwest Nigeria (latitude 7° 48′N and longitude 4°37′E). The locations are characterized by a bimodal pattern of rainfall with an annual mean of about 1300 mm with a mean temperature of 27°C and the climate is of the subhumid type. The sites had been under continuous cultivation for many years with maize; the soil at the two locations is alfisols which belongs to IWO series.

### 2.2. Treatment Application

The treatment involved three levels of poultry manure (0.0, 4.0, and 8.0 *t* ha ^−1^) and four sets of periods of urea application: (i) 0 kg N ha^−1^ (control), (ii) 120 kg N ha^−1^ applied at planting, (iii) two split applications of 120 kg N ha^−1^ (90 kg N ha^−1^ applied at planting + 30 kg N ha^−1^ at thirty days after planting), and (iv) three split applications of 120 kg N ha^−1^ (60 kg N ha^−1^ applied at planting + 30 kg N ha^−1^ thirty days after planting (DAP) + 30 kg N ha^−1^ at tasseling). These were combined to have 12 treatments, namely, (a) 0 U + 0 PM, (b) 0 U + 4 *t* ha^−1^ PM, (c) 0 U + 8 *t* ha^−1^ PM, (d) 120 kg ha^−1^ U + 0 PM, (e) 120 kg ha^−1^ U + 4 *t* ha^−1^ PM, (f) 120 kg ha^−1^ U + 8 *t* ha^−1^ PM, (g) 90 kg ha^−1^ U at planting + 30 kg ha^−1^ U at 30 DAP + 0 PM, (h) 90 kg ha^−1^ U at planting + 30 kg ha^−1^ U at 30 DAP + 4 *t* ha^−1^ PM, (i) 90 kg ha^−1^ U at planting + 30 kg ha^−1^ U at 30 DAP + 8 *t* ha^−1^ PM, (j) 60 kg ha^−1^ U at planting + 30 kg ha^−1^U at 30 DAP + 30 kg ha^−1^ U at 30 Tasseling (TAS) + 0t ha^−1^ PM, (k) 60 kg ha^−1^ U at planting + 30 kg ha^−1^ U at 30 DAP + 30 kg ha^−1^ U at 30 TAS + 4 *t* ha^−1^ PM, (l) 60 kg ha^−1^ U at planting + 30 kg ha^−1^ U at 30 DAP + 30 kg ha^−1^ U at 30 TAS + 8 *t* ha^−1^ PM.

The 12 treatments were arranged in a randomized complete block design and replicated three times.

#### 2.2.1. Land preparation, Field Layout, and Sowing of Maize Seeds

The experimental area at both sites was ploughed and harrowed. The field layout was demarcated immediately after harrowing using rope, pegs, and tape. Each experimental unit has a dimension of 5 m × 2 m. Plots were separated by 0.5 m apart, while blocks were separated by 1m apart at the two sites. The PM used was from the layers (cage system) unit of a poultry farm. The PM was composted for 2 weeks to allow for mineralization. The composted PM was weighed and spread on plots at the required rates of 0, 4, and 8 *t* ha^−1^. Rakes were used to spread the PM evenly on plots which were then incorporated to the soil to the depth of approximately 10 cm using shovels. The PM was left for 2 weeks in the soil before sowing of maize seeds. Maize seed (Empress 96), a late-maturing cultivar, was obtained from the Government Economic Service (GES) in Osogbo, Osun State. The seeds were sown at both sites on the 7th of August 2014 at a depth of 2-3 cm. Two seeds were sown per hole at a spacing of 75 cm × 25 cm which was later at two weeks after sowing thinned to one plant per stand to achieve 53 plants per plot equivalent to about 53,333 plants per hectare. Fertilizer (urea) applications were applied as required: 0 kg N ha^−1^ (control), 120 kg N ha^−1^ applied at planting, two splits of 120N kg ha^−1^ (90 kg N ha^−1^ applied at planting + 30 kg N ha^−1^ at thirty days after planting), and three splits of 120 N kg ha^−1^ (60 kg N ha^−1^ applied at planting + 30 kg N ha^−1^ thirty days after planting + 30 kg N ha^−1^ at tasseling). The first urea application was applied at planting (7th of August 2014), the second application was on the 6th and 7th of September 2014, and the third application was applied at tasseling. Fertilizer was applied by side placement about 8–10 cm away from the base of the plant. Weeds were controlled manually at three weeks interval using a hoe.

#### 2.2.2. Soil and Poultry Manure Analysis

Surface soil samples (0–15 cm depth) were collected randomly from the field at the two sites for physical and chemical analysis before the start of the experiment. The soil sample was air-dried, sieved through 2 mm sieve, and kept for analysis. The sand, silt, and clay contents were determined by hydrometer method [15]. The soil pH was determined using the pH-meter in a 1 : 2.5 soil/water ratio, total nitrogen content was by the micro-Kjeldahl method [16], available phosphorus was by Bray 1 method [17], calcium (Ca) and magnesium (Mg) were determined by the Atomic Absorption Spectrophotometer (AAS), and potassium (K) and sodium (Na) by flame emission photometry (Association of Official Analytical Chemists) [18]. The organic carbon was according to Walkey and Black using the dichromate wet oxidation method [19] and the present organic matter was estimated by multiplying the percent organic carbon with a factor 1.724. At the end of the experiment at both sites, soil samples were also collected (on a plot basis) and similarly analyzed for soil chemical properties as described above. The PM used was dried and sieved with a 2 mm sieve for chemical analysis to determine its nutrient composition. Poultry manure was analyzed for organic C, N, P, K, Ca, and Mg as described by [20].

### 2.3. Determinations of Growth and Yield Parameters

Ten (10) maize plants were used as a sample in each plot. Data collected at fortnight intervals starting from the third week after sowing included plant height, number of leaves, stem girth, and leaf area. Plant height was measured with tape from the base of the plant to the first tassel, leaf numbers were counted, leaf area was calculated using a nondestructive analysis method as suggested by [21], and stem girth was measured using a vernier caliper. Maize was allowed to dry before harvest. Yield parameters collected at harvest of maize included the weight of grain/plant and weight of cob/plant.

### 2.4. Statistical Analysis

Data on growth and yield parameters were subjected to analysis of variance (ANOVA) using the Statistical Analysis System (SAS) and means were separated using Duncan's multiple range test at *P* < 0.05.

## 3. Results

### 3.1. Soil Fertility Status of the Sites

The fertility status of the sites (I and II) in 2014 is presented in Table 1. The experimental sites are slightly acidic and sandy loam in texture. Site II has lower sand and greater percentages of silt and clay relative to Site I. Soils at both sites were low in organic matter (OM), N and P. The exchangeable bases K, Ca, and Mg were adequate according to the critical level of 3.0% OM, 0.2.0% N, 10.0 mg kg^−1^ P, 0.16–0.20 cmol kg^−1^ K, 2.0 cmol kg^−1^Ca, and 0.40 cmol kg^−1 ^Mg recommended for crop production in the agroecological zone [22]. Chemical analysis of poultry manure used indicates that it contains nutrient elements (N, P, K, Ca, Na, and Mg) required for the growth of a cereal crop such as maize.

#### 3.1.1. Effect of Different Rates of Poultry Manure and Split Application of N Fertilizer on Soil Chemical Properties

The effects of different rates of poultry manure and slit application of urea fertilizer on soil chemical properties from the two sites are presented in Table 2. At both sites, without application of urea fertilizer, PM alone increased soil pH, OM, N, P, K Ca, and Mg concentrations compared with the control (no application of PM). The increase was from 0 to 8 *t* ha^−1^PM; however, there were no significant differences between 4 and 8 *t* ha^−1^ PM for soil pH, OM, and N for Site I and pH, P, K, and Ca for Site II. Results revealed that when the effect of split application of urea fertilizer was considered at a fixed (zero) rate of PM, split applications of N fertilizer three times (60 + 30+30) has the highest values of pH, OM, N, P, K, Ca, and Mg except in Site II, where two split applications (90 + 30) have the highest value of OM. With combinations of poultry manure with urea fertilizer, split applications of N fertilizer three times (60 + 30+30) with 8 *t* ha^−1^PM (60 + 30+30 + 8 *t* ha^−1^PM) have the highest values of soil chemical properties. There were no significant differences in most cases between 60 + 30+30 + 8 *t* ha^−1^PM and 60 + 30+30 + 4 *t* ha^−1^PM at both sites.

#### 3.1.2. Effect of Different Rates of Poultry Manure and Split Application of N Fertilizer on Growth and Yield of Maize

The results of different rates of poultry manure and split application of N fertilizer on growth and yield of maize are presented in Table 3. At both sites, poultry manure alone increased the growth and the yield of maize compared with the control. Growth and yield parameters were increased from 0 to 8 *t* ha^−1^PM. Also considering the split application of urea fertilizer alone without poultry manure, application of urea fertilizer increased the growth and yield of maize compared with the control; split applications of N fertilizer three times (60 + 30+30) have the highest values of growth and yield parameters. When urea fertilizer and poultry manure are considered together; split applications of N fertilizer three times (60 + 30+30) with 8 *t* ha^−1^PM (60 + 30+30 + 8 *t* ha^−1^PM) have the highest values of growth and yield parameters of maize. There were no significant differences in most cases between 60 + 30+30 + 8 *t* ha^−1^PM and 60 + 30+30 + 4 *t* ha^−1^PM at both sites. Integrating urea with poultry manure increased growth and yield of maize compared with the sole forms of either N fertilizer or poultry manure.

## 4. Discussion

The poultry manure increased soil pH, organic matter (SOM), and N, P, K, Ca, and Mg contents of the soil compared with control. This result was expected because the soil fertility was low (Table 1). These nutrients were released into the soil over the decomposition of poultry manure. Studies by [23] and [8] have shown that poultry manure increased soil OM, N, P, K, Ca, Mg, and CEC. Poultry manure significantly increased soil pH compared with the control due to its liming effect [8]. The presence of ammonia (ammonium) in the PM also helps to increase soil pH [24].

Application of N fertilizer increased soil OM, N, P, and Mg contents of the soil relative to the control. These increases in soil OM, N, P, and Mg could probably be due to increased microbial activities as a result of N application which led to enhanced production and mineralization of OM from the soil [25]. Split application of N fertilizer three times (60 + 30 + 30) significantly had the highest values of nutrients in the soil after the experiment. This was adduced to proper application/time scheduling that minimizes N losses. Split application of N fertilizer three times (60 + 30 + 30) ensures that only a small amount of nutrients applied is lost. Mobile nutrient like N is highly soluble and is not adsorbed on clay complex especially in coarse-textured soils high in the sand as in the case of the study sites. In such soils, loss of N by leaching will be very high coupled with the high rainfall of the area (1300 mm). The authors in [26] also reported NO3 – leaching losses of up to 56 kg N ha^–1^ y^−1^ with a rainfall of 1395 mm. For such soils, during heavy rainfall, when the amount of moisture in the soil is greater than its field capacity, water will drain rapidly through the soil and leach any soluble nutrient element [27]. The combination of the sandy soil and high rainfall regime results in N from any fertilizer being highly vulnerable to leaching. Therefore, leaching loss is related to the time of application of fertilizer, soil permeability, and quality of rainfall in the ecological zone [28]. It had been reported that nitrogen and phosphorus losses from soil increase during rainy seasons when precipitation and runoff were large [29]. Split applications of N fertilizer three times (60 + 30 + 30) with 8 *t* ha^−1^PM (60 + 30+30 + 8 *t* ha^−1^PM) have the highest soil chemical properties. This can be a result of the combination of attributes of reduced nutrient loss through leaching by 60 + 30 + 30 and maximum availability of nutrients by PM at 8 *t* ha^−1^ due to an increase in organic matter from the PM.

The increase in growth and yield of maize in this study as a result of PM alone, 60 + 30 + 30 split N fertilizer applications alone, and split applications of N fertilizer three times (60 + 30 + 30) with 8 *t* ha^−1^PM (60 + 30 + 30 + 8 *t* ha^−1^PM) was due to an increase in nutrient status of the soil. For PM alone, a low C : N ratio or high N content of the PM is also important in this regard. References [8, 23] also reported that poultry manure increased the growth and yield of cocoyam (*Xanthosoma sagittifolium*) and green amaranth (*Amaranthus hybridus*), respectively. For, 60 + 30 + 30 split N fertilizer alone, it is suggested that there was better availability of soil nutrients for plant use when fertilizer was split-applied three times compared with other methods [13]. The growth and yield of maize under poultry manure alone, urea fertilizer alone, and the combinations of urea fertilizer with poultry manure are consistent with the soil chemical properties of these treatments. Hence, soil chemical properties were significantly correlated with growth and yield of maize in this experiment (Table 4).

Compared with split application methods, the values of growth and yield of maize under120 kg N ha^−1^ applied once were low because the N applied at planting was more susceptible to leaching losses at the time when the plant was still young and N uptake rate was very low [30]. Maize plant needs about 2-3 weeks after sowing to develop leaves and roots needed for nutrient absorption. The seminal roots develop after the germination of maize seed is not yet absorbing nutrients. The seedlings at this stage depend solely on the kernel food reserve [31, 32]. Any fertilizer applied at planting will, therefore, be subjected to leaching loss since absorption by the maize up to 10–15 days after sowing maize seed is rare [30]. Sitthaphanit et al. [14] also reported that maize grain yield increased with the split application of fertilizer compared to one single application at planting. The rate of utilization of N gradually increased and reached maximum just before tasseling [33, 34]. During this stage (just before tasseling), N utilization by maize could be as high as 4 kg N ha day^−1^ [35]. Sharifi and Namvar [35] reported that maize yield increased when N was applied up to the tassel stage. Therefore, for better growth and yield of maize, N fertilizer application should be structured in accordance with this pattern of uptake to avoid losses by leaching and therefore ensured that N level in the soil is high at the critical stage of N demand. Maintaining a substantial level of N in the soil during tasseling is important in spikelet differentiation and also for kernel formation [13, 36]. Bhattarai et al. [37] also reported that the application of N fertilizer at equal doses of 60 kg ha-1 at sowing, earthing up, and silking stages maximized the yield of maize.

Results showed that maize yielded higher under N fertilizer integrated with poultry manure. This could be attributed to increased nutrient use efficiency following the inclusion of the N fertilizer with poultry manure [38, 39]. It can be said that the addition of N fertilizer to poultry manure aided mineralization of nutrients in poultry manure due to enhanced supply of nutrients, leading to better growth and yield. In addition, poultry manure may have prevented the leaching of N fertilizer by improving soil structure and quality as well as by unspecific binding of mineral nutrients [40]. This study agrees with the findings of [41] who reported that the most satisfactory method of increasing maize yield was by combining the application of organic wastes and inorganic fertilizer. Authors in [42] also reported better performance of maize, cassava, and melon under poultry manure + NPK fertilizer.

This research work supports the findings of [43, 44] that integrated nutrient application gave higher crop yield compared with the recommended sole inorganic and organic fertilizers. The authors also reported that an integrated application of organic and inorganic fertilizer would build up soil productivity and quality on a long-term basis. The presence of poultry manure helps the soil to maintain higher rate of applied urea intact or in the form of ammonium ions for a longer time, resulting in higher nitrogen uptake efficiency [45, 46]. Similarly, it has been shown that integration or combination of mineral N with organic materials helps to increase soil N use efficiency probably due to acting as a simple controlled release fertilizer or reduction in N mineralization [47, 48].

Therefore, for better growth and yield of maize, after the initial application of poultry manure, N fertilizer application should be structured by split applications of the fertilizer three times in accordance with the pattern of uptake to avoid losses by leaching and therefore ensured that N level in the soil is high at the critical stage of N demand. However, because the yield between 4 and 8 *t* ha^−1^PM in combination with 60 + 30+30 urea fertilizer was statistically similar and due to the bulkiness, handling challenges, and cost of poultry manure, 4 *t* ha^−1^PM with the split application of urea fertilizer three times (60 + 30 + 30) is recommended.

## 5. Conclusion

Results of this experiment showed that PM, N (urea) fertilizer (either applied once at planting or split-applied), and integrating N fertilizer with poultry manure improved soil chemical properties, growth, and yield of maize compared with the control. Application of 60 + 30 + 30 resulted in the most improved soil chemical properties, growth, and yield of maize. Results also showed that maize yielded higher under N fertilizer integrated with poultry manure compared with their sole forms. Application of 60 + 30 + 30 + 8 *t* ha^−1^PM or those with 4 *t* ha^−1^PM treatments showed the highest growth and yield of maize and, therefore, 60 + 30 + 30 + 4 *t* ha^−1^PM is recommended. In addition, for better growth and yield of maize, after the initial application of poultry manure, N should be split-applied in accordance with plant growth and the pattern of uptake to avoid losses by leaching and therefore ensured that N level in the soil is high at the critical stage of N demand.

## Figures and Tables

**Table 1:** Initial soil characteristics at the sites before experimentation.

Property	Site I	Site II	Poultry manure
Sand (%)	82.4	61.4	ND
Silt (%)	3.28	17.3	ND
Clay (%)	14.32	21.3	ND
Textural class	Sandy loam	Sandy loam	
Organic C (%)	1.65	1.24	1.76
pH (water)	5.78	5.60	6.80
Total N (%)	0.18	0.14	2.42
Available P (mg kg^−1^)	6.56	5.46	4.20
Exchangeable K (cmol kg^−1^)	0.36	0.48	0.48
Exchangeable Ca (cmol kg^−1^)	2.10	2.30	0.64
Exchangeable Mg (cmol kg^−1^)	0.75	0.50	0.34

**Table 2:** Effects of poultry manure and split urea fertilizer on soil chemical properties.

Split urea fertilizer (kg ha^−1^)	Poultry manure rate (t·ha^−1^)	pH (water)		OM (%)		N (%)		P (mg kg^−1^)		K (cmol kg^−1^)		Ca (cmol kg^−1^)		Mg (cmol kg^−1^)	
		Site I	Site II	Site I	Site II	Site I	Site II	Site I	Site II	Site I	Site II	Site I	Site II	Site I	Site II
Control	0	5.70b	5.59b	2.08e	2.02g	0.14e	0.13i	6.50d	4.22c	0.18h	0.26c	0.76d	2.12f	0.50c	0.43h
	4	5.84a	6.71a	2.89b	3.50b	0.16d	0.14hi	8.96b	4.85a	0.20fg	0.52a	1.15b	2.42d	0.57b	1.64b
	8	5.89a	6.78a	2.99b	3.60a	0.16d	0.15gh	9.48a	4.86a	0.25d	0.53a	1.25a	2.42d	0.67a	1.65b

120	0	5.48e	5.59b	2.18cd	2.21e	0.16d	0.20f	6.51d	4.68b	0.19gh	0.29b	0.98c	2.36e	0.49c	0.82g
	4	5.52d	6.65a	2.91b	2.92c	0.18c	0.22e	9.84a	4.89a	0.20e	0.53a	1.18b	2.57c	0.58b	0.84g
	8	5.58d	6.71a	3.01ab	2.30d	0.19bc	0.23d	9.90a	4.89a	0.27c	0.54a	1.26a	2.58c	0.69a	1.20e

90 + 30	0	5.64cd	5.58b	2.21cd	2.20ef	0.18c	0.25c	6.53d	5.25b	0.28c	0.30b	0.77d	2.45d	0.49c	0.99f
	4	5.74b	6.48a	3.07a	3.63a	0.19bc	0.27b	7.70cd	5.77a	0.31ab	0.54a	1.15b	2.78b	0.58b	1.34d
	8	5.76b	6.48a	3.15a	3.68a	0.20b	0.29ab	8.06b	5.79a	0.33a	0.54a	1.17b	2.78b	0.68a	1.64b

60 + 30+30	0	5.74b	5.60b	2.22cd	2.18f	0.19bc	0.29ab	6.60d	5.25b	0.29b	0.32b	0.79d	2.45d	0.50c	1.44c
	4	5.86a	6.44a	3.09a	3.67a	0.22ab	0.31a	9.98a	5.79a	0.32a	0.56a	1.28a	2.86a	0.61ab	1.94a
	8	5.88a	6.48a	3.11a	3.69a	0.23a	0.31a	9.99a	5.80a	0.32a	0.56a	1.29a	2.79b	0.61ab	1.94a

Values followed by similar letters under the same column are not significantly different at *P* = 0.05 according to Duncan's multiple range test.

**Table 3:** Effects of poultry manure and split urea fertilizer on growth and yield of maize.

Split urea fertilizer (kg ha^−1^)	Poultry manure rate (t ha^−1^)	Plant height (cm)		Number of leaves/plant		Leaf area (cm^2^)		Number of grains/cob		Weight of cob (g)		Weight of 1000 grains (g)	
		Site I	Site II	Site I	SiteII	Site I	Site II	Site I	Site II	Site I	Site II	Site I	Site II
Control	0	118.8e	127.5e	10.1d	9.2c	451.1j	557.4g	434.3g	240.1g	153.8e	94.8i	324.0e	259.6g
	4	166.8d	131.1d	11.5bc	11.4b	677.6i	652.3f	476.9ef	354.6f	160.2cd	124.9h	343.1d	278.8f
	8	198.2c	143.1cd	11.9bc	11.4b	751.4h	675.8ef	499.4ef	409.53	164.6cd	132.9f	358.9cd	328.9e

120	0	192.0c	146.7cd	12.1bc	11.2b	802.1g	735.1d	456.8fg	488.8c	159.1cd	134.4f	357.3cd	327.3e
	4	198.6c	136.5d	12.4ab	11.0b	843.6fg	774.2d	473.7ef	439.7d	168.5cd	147.2e	365.1bc	359.1d
	8	202.4bc	153.8bc	12.5ab	11.2b	884.9ef	874.6bc	525.6d	488.2c	177.5bc	149.8e	369.4bc	364.4d

90 + 30	0	210.8bc	151.2bc	12.2ab	12.2a	841.1fg	804.5c	527.8d	522.3b	175.1bc	161.8d	359.8cd	359.8d
	4	214.1b	155.2bc	12.6ab	11.8b	931.6de	863.5bc	559.1cd	548.4b	186.8ab	175.8cd	369.0bc	360.0d
	8	220.5b	159.9b	12.7ab	11.2b	979.4cd	880.7bc	620.4cd	505.4b	189.9b	177.3c	374.1b	374.1c

60 + 30+30	0	218.0b	154.0bc	12.7ab	12.5a	1006.0c	804.5c	660.8bc	600.0b	197.1a	190.8ab	357.3cd	359.3b
	4	223.6ab	164.8ab	12.8a	12.4a	1120.1b	929.1ab	702.8ab	614.0ab	198.4a	194.1ab	404.5a	404.5a
	8	230.2a	170.3a	13.1a	12.0a	1185.2a	986.0a	712.3a	654.8a	205.6a	204.8a	416.6a	416.6a

Values followed by similar letters under the same column are not significantly different at *P*=0.05 according to Duncan's multiple range test.

**Table 4:** Correlation coefficient between growth and yield parameters of maize and soil chemical properties (means of Site I and Site II).

	Plant height	Number of leaves/plant	Leaf area	Number of grains/cob	Weight of cob	Weight of 1000 grains
pH	0.25	0.24	0.28	0.15	0.20	0.32
OM	0.46	0.40	0.48	0.38	0.43	0.51
N	0.91^*∗∗*^	0.82^*∗∗*^	0.97^*∗∗*^	0.95^*∗∗*^	0.97^*∗∗*^	0.92^*∗∗*^
P	0.54^*∗*^	0.58^*∗*^	0.62^*∗*^	0.58^*∗*^	0.57^*∗*^	0.69^*∗*^
K	0.67^*∗*^	0.59^*∗*^	0.70^*∗*^	0.58^*∗*^	0.64^*∗*^	0.70^*∗*^
Ca	0.70^*∗*^	0.61^*∗*^	0.75^*∗∗*^	0.60^*∗*^	0.63^*∗*^	0.78^*∗∗*^
Mg	0.70^*∗*^	0.61^*∗*^	0.75^*∗∗*^	0.60^*∗*^	0.63^*∗*^	0.78^*∗∗*^

^*∗*^Significant difference at *P*=0.05; ^*∗∗*^ significant difference at *P*=0.01

## Data Availability

All data used to support the findings of this study are included within the article.
